# Retrospective study of the differential diagnosis between cryptogenic multifocal ulcerous stenosing enteritis and small bowel Crohn’s disease

**DOI:** 10.1186/s12876-020-01389-7

**Published:** 2020-08-05

**Authors:** Dan Chen, Wei Liu, Weixun Zhou, Weiyang Zheng, Dong Wu, Jiaming Qian

**Affiliations:** 1 Department of Gastroenterology, Peking Union Medical College Hospital, Chinese Academy of Medical Sciences and Peking Union Medical College, Shuaifuyuan, No.1, Dongcheng District, Beijing, 100730 China; 2 Department of Radiology, Peking Union Medical College Hospital, Chinese Academy of Medical Sciences and Peking Union Medical College, Beijing, China; 3 Department of Pathology, Peking Union Medical College Hospital, Chinese Academy of Medical Sciences and Peking Union Medical College, Beijing, China

**Keywords:** Cryptogenic multifocal ulcerous stenosing enteritis, Small bowel Crohn’s disease, Small intestinal ulcer, Differential diagnosis

## Abstract

**Background:**

Being a rare disease, cryptogenic multifocal ulcerous stenosing enteritis (CMUSE) is easily misdiagnosed as small bowel Crohn’s disease (SBCD).

**Aims:**

This study was aimed to compare clinical features of CMUSE to SBCD.

**Methods:**

Fourteen patients with CMUSE and 61 patients with SBCD were retrospectively analyzed.

**Results:**

Hematochezia was more frequent in CMUSE patients (10, 71.4% vs 23, 37.7%, *P* = 0.022), while diarrhea was more common in SBCD patients (23, 37.7% vs 0, 0.0%, *P* = 0.015). More patients with CMUSE developed intestinal stenosis than with SBCD (14, 100% vs 37, 60.7%, *P* = 0.011). 30 (50.0%) SBCD patients and none CMUSE patients had an elevated erythrocyte sedimentation rate level (*P* = 0.001). Extra-enteric findings found by computed tomography enterography were significantly more prevalent in SBCD patients than in CMUSE patients (25,71.4% vs 3,25%, *P* = 0.013). Longitudinal ulcers found by endoscopy were more common in SBCD patients (16, 37.2% vs 0, 0.0%, *P* = 0.041), while circumferential ulcers were more common in CMUSE patients (6, 54.6% vs 8, 18.6%, *P* = 0.041). All ulcers observed in CMUSE patients were within mucosal and submucosal layers, but 8 (44.4%) SBCD patients had deep ulcers that reached beyond submucosal layers (*P* = 0.003). Ulcers were located at strictures in 9 (90.0%) CMUSE patients but only in 1 (5.6%) SBCD patient (*P* = 0.000).

**Conclusions:**

Gastrointestinal symptoms, erythrocyte sedimentation rate levels, radiologic, endoscopic and pathologic features help to distinguish CMUSE from SBCD.

## Background

Cryptogenic multifocal ulcerous stenosing enteritis (CMUSE) is a rare condition affecting the small bowel, characterized by multiple unexplained ulcerations and strictures without systemic inflammation typically, but not always, in middle-aged or young patients. The etiology is unknown and pathogenesis is poorly understood. Abdominal pain, ileus and hematochezia are its common symptoms. Extra-intestinal manifestations of CMUSE include oral aphthae, joint pain, Raynaud’s phenomenon, sun allergy and neuropathy [1].

Crohn’s disease (CD) is an intestinal inflammatory disease but can affect the entire gastrointestinal tract. Small bowel is involved in almost 80% of the cases [2]. Small bowel Crohn’s disease (SBCD) is defined as CD with lesions mainly located in the small bowel which occur in as many as one third of CD patients [3]. Typical presentations include the presence of longitudinal ulcers with a cobblestone appearance, skip lesions, and the development of complications such as strictures and fistulas. Oral ulcers, arthralgia, erythema nodosum, and autoimmune hepatic diseases are its extra-intestinal manifestations.

Less than 100 cases of CMUSE have been published worldwide since it was first described by Debray et al. in 1964 [4]. A retrospective study in Korea reported that 90% CMUSE patients had been misdiagnosed with SBCD [5]. Both CMUSE and SBCD are chronic recurrent diseases, with similar gastrointestinal symptoms and multiple ulcerations and strictures of the small intestine. Distinguishing between them is challenging but important, since the treatment and prognosis of CMUSE differs from SBCD [3, 6]. To our knowledge, there are just 3 case reports [7–9] and 2 case series [1, 10] of CMUSE proposing some points for differentiation. No comparative study regarding the differential diagnosis between CMUSE and SBCD has been done so far. Therefore we conducted this retrospective study to investigate clinical features of CMUSE and SBCD to find out the main points beneficial for differentiation between them.

## Methods

### Patients

This was a retrospective study in a single center. The medical records of 14 patients with CMUSE from August 1984 to March 2017 in Peking Union Medical College Hospital, Beijing, China were reviewed. The control group of 61 patients with small bowel Crohn’s disease (SBCD) was selected from our IBD database using the simple random method with the procedure of “SURVEYSELECT” of SAS package. All patients were followed up for at least 12 months. Patients diagnosed with Crohn’s disease with colorectal lesions, ulcerative colitis, intestinal tuberculosis, Behcet’s disease, neoplasms, or other recognized causes of intestinal inflammation were excluded.

### Diagnostic criteria of small bowel Crohn’s disease

A diagnosis of SBCD was established by clinical evaluation and a combination of endoscopic, histological, radiological, and/or biochemical investigations according to 3rd European Evidence-based Consensus on the Diagnosis and Management of Crohn’s Disease 2016 [11].

### Diagnostic criteria of cryptogenic multifocal ulcerous stenosing enteritis

The diagnostic criteria for CMUSE were [5]: (1) unexplained small bowel strictures or ileus; (2) superficial ulcer in the mucosa and submucosa; (3) chronic or relapsing ulcerative stenosis of the small bowel after surgical resection; (4) no signs of systemic inflammation; (5) improvement after administering corticosteroids; (6) excluding other small intestine ulcerative disease. We recommended that a complete CMUSE be diagnosed if pathologic findings show that superficial ulcer is limited within mucosal and submucosal layers, and a suspected CMUSE be diagnosed if lacking of surgical resection and pathologic examination could not been done. In this study, 10 (71.4%) and 4 (28.6%) patients were diagnosed as complete and suspected CMUSE, respectively. In fact, seven CMUSE patients had been misdiagnosed with CD, and one with intestinal tuberculosis, Meckel diverticulum and ischemic bowel disease, respectively.

### Clinical, laboratory, radiologic, endoscopic, and pathologic features

Demographics (gender, age at gastrointestinal symptom onset and disease duration), the history of appendectomy, medication history of nonsteroidal anti-inflammatory drugs (NSAIDs) for more than 2 weeks, body mass index (BMI), clinical manifestations (symptoms, extra-intestinal manifestations, gastrointestinal complications, and lesion distribution), laboratory findings, radiologic features, endoscopic abnormalities, and pathologic features were collected. Haematochezia is defined as overt bloody stool. Lesion distribution was determined by radiologic and endoscopic examinations and surgical operation. Terminal ileum was defined as ileum that was within 30-cm of ileocecal valve. Radiologic features included skip lesions, enteric findings (ulcers, bowel strictures, bowel wall thickening, mural hyperenhancement, rough serosa, pseudodiverticulum and pseudo-polyps) and extraenteric findings (comb sign, enhanced density of the peri-intestinal fat, enlargement of the abdominal lymph nodes, and fistula) on CTE. Endoscopic features included the presence and number of ulcers (1, or ≥ 2), ulcer types (longitudinal ulcer, dot ulcer, circumferential ulcer, oval ulcer, aphthous ulcer or irregular ulcer), number of strictures (1, or ≥ 2), fistula, hyperplastic lesions (pseudo-polyps, nodular lesions or eminence lesions) and cobblestone appearance detected by double-balloon enteroscopy, coloscopy or capsule endoscopy. Aphthous ulcer was defined as a tiny, punched out, raised or flat red lesion with a white center.

Operation data included the number of patients underwent operation and the surgical indications. All samples for histopathological examination were surgical samples. Pathological feature included ulcer incidence, ulcernumber (1, or ≥ 2), the depth of the deepest ulcer (deep, or superficial, superficial ulcer means ulcer limited within mucosal and submucosal layers), ulcer located on stricture site, stricture number (1, or ≥ 2), cobblestone appearance, perforation, fistula, transmural inflammation, non- caseous epithelioid granulomas, and abscess. Images of radiologic, endoscopic or pathologic examinations were respectively reviewed by a radiologist (Wei Liu), an endoscopic expert (Weiyang Zheng) or pathologist (Weixun Zhou) who was blinded to the design of this study.

### Statistical analysis

SPSS 22.0 was used for data analysis. Continuous variables were presented as mean ± standard deviation (SD) or medians with interquartile range. Differences in quantitative data between the two groups were analyzed by independent t-test for continuous variables followed normal distribution or by non-parametric test for continuous variables not coincided with normal distribution. Chi-squared test or Fisher’s exact test were used for categorical variables. *P* values were two-tailed, and *P* value of < 0.05 was considered to be statistically significant.

## Results

### Demographics and clinical features

Table 1 shows demographics and some clinical data of CMUSE patients and SBCD patients. No significant difference was found with respect to the patients’ gender, age, disease duration, the history of appendectomy and BMI. No patients reported to have used NSAIDs for more than 2 weeks.

**Table 1:** Demographic and clinical features of CMUSE and SBCD patients

Characteristics	CMUSE		SBCD		*P* value
	N	n (%) or median/mean	N	n (%) or median/mean	
Demographics					
Gender (male, n (%))	14	8 (57.1)	61	34 (55.7)	0.924
Disease duration (months, median(P_25_,P_75_))	14	33.5 (11.3–168.0)	61	24.0 (5.5–84.0)	0.320
The history of appendectomy (n (%))	14	5 (35.7)	61	13 (21.3)	0.429
Medication history of NSAIDs for more than two weeks (n (%))	14	0 (0.0)	61	0 (0.0)	1.000
BMI (mean ± SD)	7	18.68 ± 1.65	49	19.25 ± 3.32	0.659
General symptoms (n (%))					
Fever	14	3 (21.4)	61	29 (47.5)	0.075
Weakness	14	8 (57.1)	61	27 (44.3)	0.384
Weight loss	14	8 (64.3)	61	48 (78.7)	0.429
Gastrointestinal symptoms (n (%))					
Poor appetite	14	6 (42.9)	61	37 (60.7)	0.225
Hematochezia	14	10 (71.4)	61	23 (37.7)	0.022
Abdominal pain	14	11 (85.7)	61	56 (91.8)	0.844
Intestinal distention	14	8 (57.1)	61	25 (41.0)	0.272
Vomiting	14	8 (57.1)	61	27 (44.3)	0.384
Diarrhea	14	0 (0.0)	61	23 (37.7)	0.015
Abdominal mass	14	4 (28.6)	61	21 (34.4)	0.917
Perianal lesions	14	1 (7.1)	61	3 (4.9)	0.571
Extra-intestinal manifestations (n (%))	14	3 (23.1)	61	28 (45.9)	0.130
Oral ulcers	14	2 (14.3)	61	21 (34.4)	0.249
Genital ulcers	14	0 (0.0)	61	3 (4.9)	1.000
Joint pain	14	0 (0.0)	61	11 (18.0)	0.139
Skin lesions	14	1 (7.1)	61	5 (8.2)	1.000
Gastrointestinal complications (n (%))					
Perforation	14	0 (0.0)	61	2 (3.3)	1.000
Fistula	14	0 (0.0)	61	5 (8.2)	0.607
Intestinal stenosis	14	14 (100.0)	61	37 (60.7)	0.011
Abscess	14	0 (0.0)	61	3 (4.9)	1.000
Disease distribution (n (%))					
Jejunum	14	4 (28.6)	61	27 (44.3)	0.282
Ileum	14	13 (92.9)	61	57 (93.4)	1.000
Upper gastrointestinal tract	14	1 (7.1)	61	6 (9.8)	1.000
Terminal ileum	14	4 (28.6)	61	38 (62.3)	0.022
Ileocecal valve	14	2 (14.3)	61	12 (19.7)	0.931

*CMUSE* cryptogenic multifocal ulcerous stenosing enteritis; *SBCD* small bowel Crohn’s disease; *NSAIDs* non-steroidal antiinflammatory drugs; *BMI*: body mass index

Abdominal pain was not only the most common reporting symptom (8, 57.1% vs 36, 59.0%, *P* = 0.898) but also the most frequent gastrointestinal symptom (12, 85.7% vs 56, 91.8%, *P* = 0.844) in both CMUSE patients and SBCD patients. Hematochezia was more frequent in CMUSE patients (10, 71.4% vs 23, 37.7%; *P* = 0.022), while diarrhea was more common in CD patients (23, 37.7% vs 0, 0.0%, *P* = 0.015, respectively). CMUSE patients (14, 100.0%) had a significantly higher incidence of developing intestinal stenosis than SBCD (37, 60.7%) (*P* = 0.011). Terminal ileum was found to be more frequently involved in SBCD patients (38, 62.3%) than CMUSE patients (4, 28.6%) (*P* = 0.022). Upper gastrointestinal tract involved in one CMUSE patient manifested as duodenal ulcer. There was no significant difference in general symptoms or extra-intestinal manifestations.

### Laboratory findings

The laboratory findings of CMUSE patients and SBCD patients are listed in Supplementary Data Content 1. CMUSE patients (12, 92.3%) were more likely to develop anemia than SBCD patients (36, 60.0%) (*P* = 0.028). The mean hemoglobin level of patients with CMUSE and SBCD were 93.2 ± 27.6 and 104.6 ± 27.0 g/L (*P* = 0.164), respectively. Besides, 30 (50.0%) of the patients with SBCD were observed to have elevated ESR levels, while none of the CMUSE patients had elevated ESR levels (*P* = 0.001). The levels of high sensitivity C reactive protein (hsCRP) were elevated in more SBCD patients (39, 68.4%) than CMUSE patients (4, 28.6%), (*P* = 0.006).

### Radiologic features

**Fig. 1:**
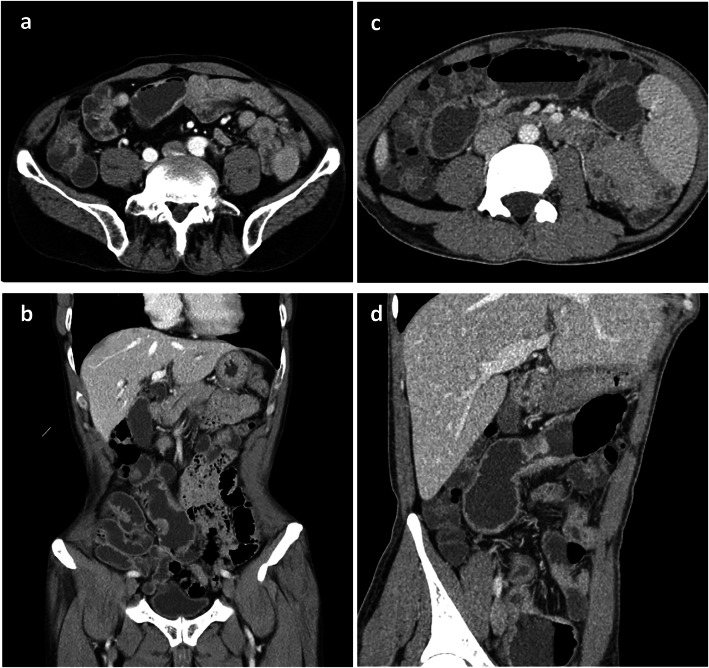
The radiologic features of CMUSE and SBCD patients. **a** and **b** were CTE images from a patient with CMUSE showing multiple mild stenosis, luminal dilatationand and mild mucosal hyperenchancement of the ileum on the right side. **c** and **d** were CTE images from two patients with SBCD which showed multiple stenosis, mucosal hyperenchancement, thickened wall, roughness of serosa, luminal narrowing of the small intestine, and the comb sign

The radiologic features (Fig. 1) found by CTE of CMUSE patients and SBCD patients are summarized in Table 2. Both groups were manifested as skip lesions on CTE. Extra-enteric findings were significantly more prevalent in SBCD patients (25, 71.4%) than in CMUSE patients (3, 25.0%) (*P* = 0.013). Specifically, enlargement of the abdominal lymph nodes was more frequently observed in SBCD patients (21, 60.0%) than CMUSE patients (2, 16.7%) (*P* = 0.010).

**Table 2:** Radiologic features of CMUSE and SBCD patients

Characteristics	CMUSE		SBCD		*P* valua
	N	n(%)	N	n(%)	
Number of patients underwent CTE	14	12 (85.7)	61	35 (57.4)	/
Skip lesions	12	8 (66.7)	35	20 (57.1)	0.811
Enteric findings	12	10 (83.3)	35	32 (91.4)	0.808
Ulcers	12	0 (0.0)	35	2 (5.7)	1.000
Bowel strictures	12	8 (66.7)	35	13 (37.1)	0.076
Bowel wall thickening	12	10 (83.3)	35	30 (85.7)	1.000
Mural hyperenhancement	12	7 (58.3)	35	29 (82.9)	0.181
Roughness of serosa	12	0 (0.0)	35	8 (22.9)	0.170
Pseudodiverticulum	12	1 (8.3)	35	5 (14.3)	1.000
Pseudo-polyps	12	0 (0.0)	35	2 (5.7)	1.000
Extra-enteric findings	12	3 (25.0)	35	25 (71.4)	0.013
Comb sign	12	1 (8.3)	35	3 (8.6)	1.000
Enhanced density of the peri-intestinal fat	12	0 (0.0)	35	5 (14.3)	0.309
Enlarged lymph nodes	12	2 (16.7)	35	21 (60.0)	0.010
Fistula	12	0 (0.0)	35	4 (11.4)	0.560

*CMUSE* cryptogenic multifocal ulcerous stenosing enteritis; *SBCD* small bowel Crohn’s disease; *CTE* computed tomography enterography

### Endoscopic features

**Fig. 2:**
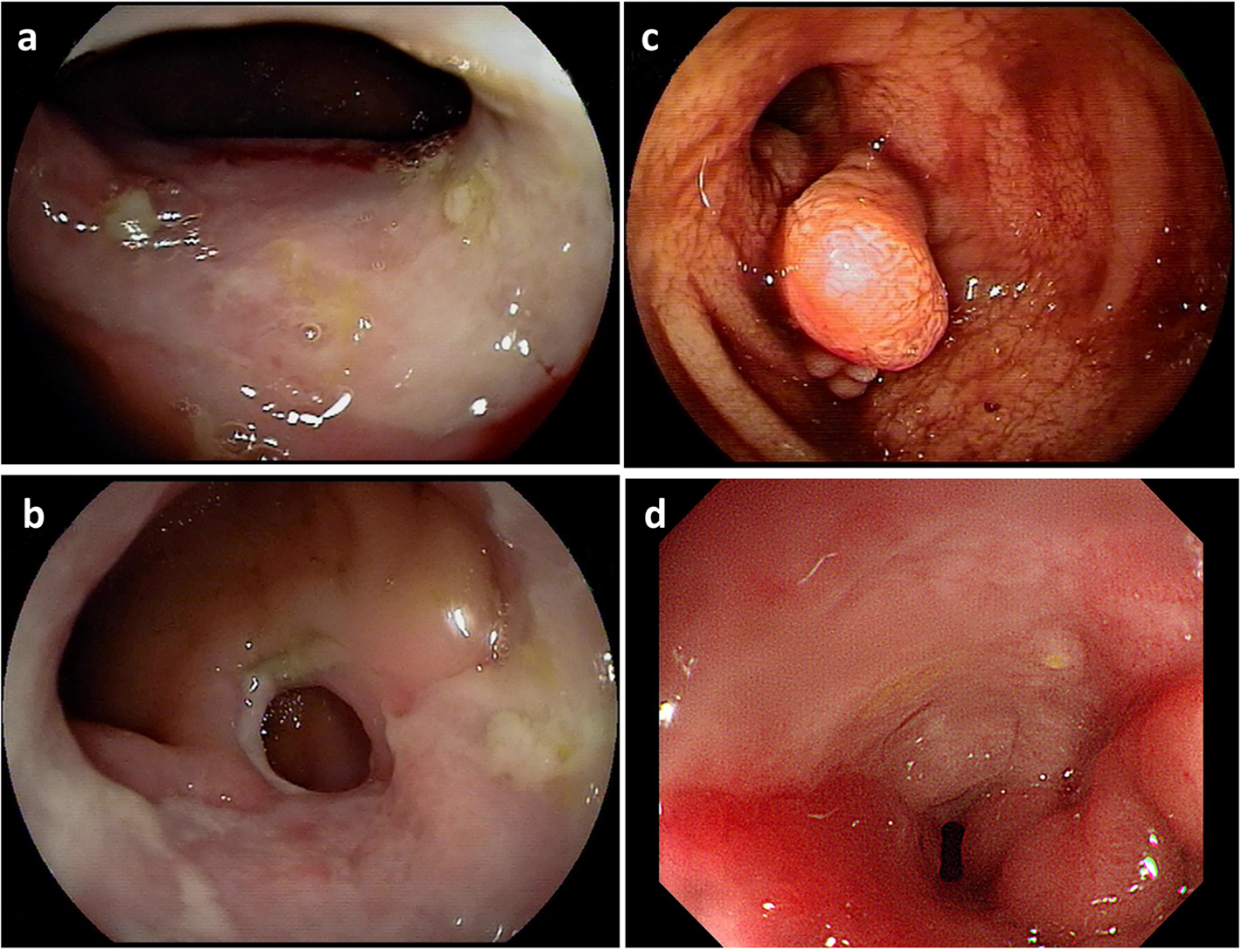
The endoscopic features of CMUSE and SBCD patients. **a** and **b** were double-balloon enteroscopy images from a patient with CMUSE showing multiple centripetal annular stenosis with circumferential ulcers located on of the fourth group small intestine. **c** was a double-balloon enteroscopy image from a SBCD patients showing luminal narrowing with multiple inflammatory polyp on anal site of the sixth group small intestine. **d** was a coloscopy image from patients with SBCD revealing a longitudinal ulcer with cobblestone appearance on the opposite site of terminal ileum

The endoscopic features (Fig. 2) of CMUSE patients and SBCD patients are listed in Table 3. Longitudinal ulcers were more common in SBCD patients (16, 37.2% vs 0,0.0%, *P* = 0.041), while circumferential ulcers were more common in CMUSE patients (6,54.6% vs 8,18.6%, *P* = 0.041). More patients with CMUSE developed strictures than with SBCD (9, 69.2% vs 16, 29.6%, *P* = 0.020) and CMUSE patients were more likely to have multiple strictures compared to SBCD patients (4, 44% vs 3, 18.7%, *P* = 0.013). Cobblestone appearance, which was considered as the characteristic change of CD, tended to be observed in more SBCD patients (4, 7.4%) than CMUSE patients (0, 0.0%), but the differences were not significant (*P* = 1.000).

**Table 3:** Endoscopic features of CMUSE and SBCD patients

Characteristics	CMUSE		SBCD		*P* value
	N	n(%)	N	n(%)	
Number of patients taking endoscopy	14	13 (92.9)	61	54 (88.5)	/
Coloscopy	14	11 (78.6)	61	43 (70.5)	0.782
Double-balloon enteroscopy	14	12 (85.7)	61	22 (36.1)	0.001
Capsule endoscopy	14	5 (35.7)	61	15 (24.6)	0.607
Capsule retention	5	3 (60.0)	15	3 (20.0)	0.131
Gastroscopy	14	12 (85.7)	61	36 (59.0)	0.061
Lesion detected by endoscope					
Ulcer	13	11 (84.6)	54	43 (79.6)	0.986
Number of ulcers					
1	11	1 (9.1)	43	11 (25.6)	0.498
≥ 2	11	8 (72.7)	43	27 (62.8)	
Unknown	11	2 (18.2)	43	5 (11.7)	/
Ulcer type					
Longitudinal ulcer	11	0 (0.0)	43	16 (37.2)	0.041
Dot ulcer	11	2 (18.2)	43	7 (16.3)	1.000
Circumferential ulcer	11	6 (54.6)	43	8 (18.6)	0.041
Oval ulcer	11	2 (18.2)	43	7 (16.3)	1.000
Irregular ulcer	11	1 (9.1)	43	19 (44.2)	0.072
Unknown	11	1 (9.1)	43	1 (2.4)	/
Stricture	13	9 (69.2)	54	16 (29.6)	0.020
Number of strictures					
1	9	2 (22.2)	16	11 (68.8)	0.013
≥ 2	9	4 (44.5)	16	3 (18.7)	/
Unknown	9	3 (33.3)	16	2 (12.5)	
Fistula	13	1 (7.7)	54	0 (0.0)	0.194
Hyperplastic lesions	13	3 (23.1)	54	15 (27.8)	1.000
Pseudo-polyps	3	1 (33.3)	15	5 (33.3)	1.000
Nodular lesions	3	1 (33.3)	15	8 (53.3)	1.000
Eminence lesions	3	2 (66.7)	15	3 (20.0)	0.172
Cobblestone appearance	13	0 (0.0)	54	4 (7.4)	1.000

*CMUSE* cryptogenic multifocal ulcerous stenosing enteritis; *SBCD* small bowel Crohn’s disease

### Surgical operations

Operation data of CMUSE and SBCD patients including proportion of patients underwent surgery and surgical indications are summarized in Supplementary Data Content 2. 10 (71.4%) CMUSE patients and 25 (41.0%) SBCD patients underwent at least one intestinal operation (*P* = 0.039). Ileus was the most common surgical indication for both CMUSE patients (6, 60.0%) and SBCD patients (12, 48.0%) (*P* = 0.711).

### Pathologic features

**Fig. 3:**
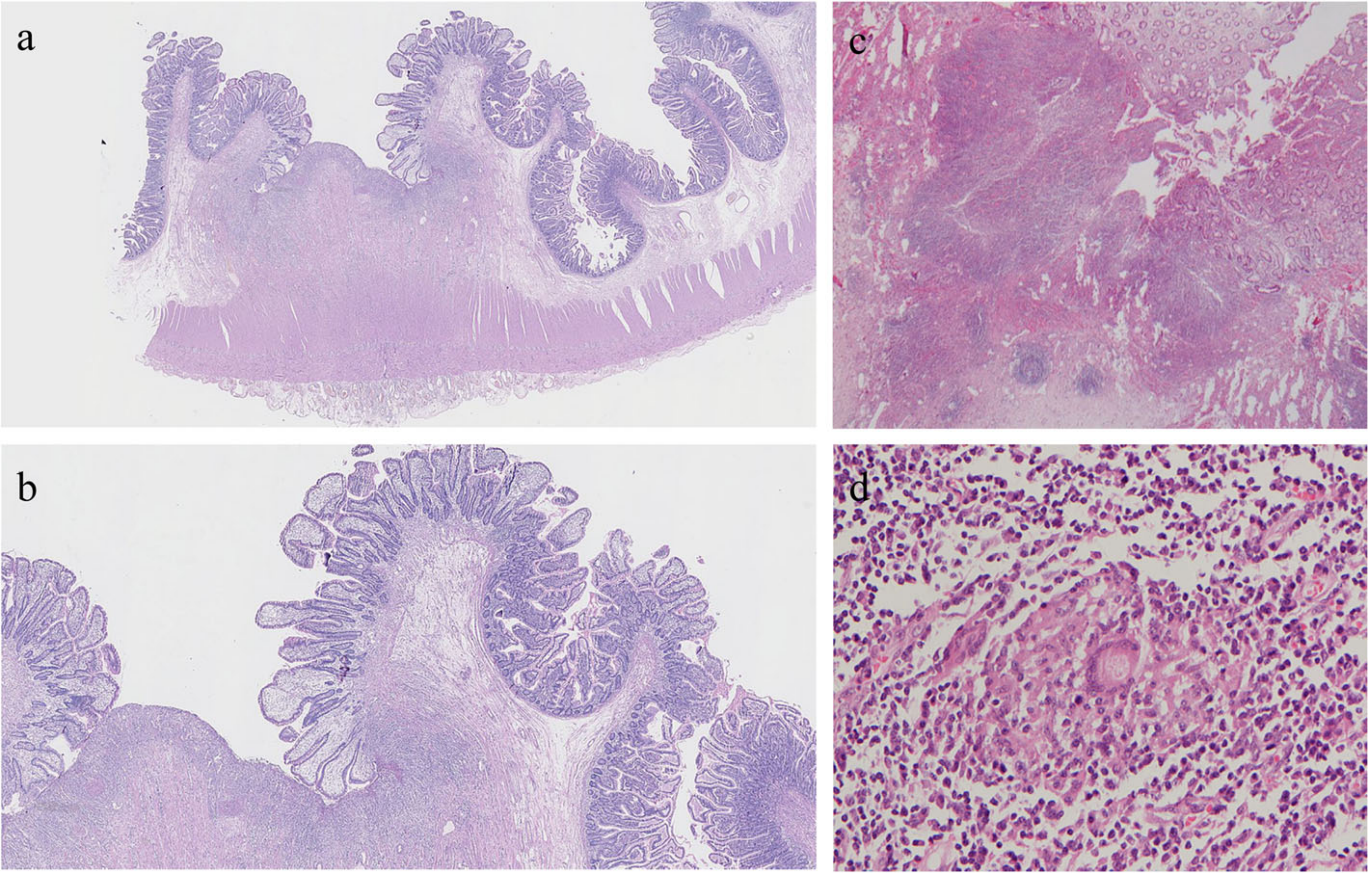
The pathologic features of CMUSE and SBCD patients. Microscopic findings on a surgical specimen obtained from a CMUSE patient stained with hematoxylin and eosin (HE) showed superficial ulcer affecting the mucosa and submucosa (**a**:4×, **b**:10×). In comparison, pathologic tissue stained with HE in patients with SBCD showed deep ulcer with transmural inflammation(**c**:10×)and non- caseous epithelioid granulomas (**d**:20×)

Superficial ulcers and strictures were found in surgery pathology in all patients with CMUSE. Ulcers and strictures in 8(80%) patients with CMUSE were multiple. Cobblestone appearance, transmural inflammation and non- caseous epithelioid granulomas were absent in all the CMUSE cases. Table 4 shows the pathologic features (Fig. 3) of CMUSE patients and SBCD patients. There were significant differences in the ulcer location (*P* = 0.000) and ulcer depth (*P* = 0.003) between the two groups. Ulcer was observed to be located on strictures in 9 (90.0%) CMUSE patients but only in 1 (5.6%) SBCD patient. All ulcers observed in CMUSE patients (10, 100%) were superficial which were limited within mucosal and submucosal layers. Eight (44.4%) SBCD patients had deep ulcers that reached out of submucosal layers. Similarly, the incidence of transmural inflammation was higher in SBCD patients (16, 64.0%) than CMUSE patients (1, 10.0%) (*P* = 0.007).

**Table 4:** Pathologic features of CMUSE and SBCD patients

Characteristics	CMUSE		SBCD		*P* value
	N	n(%)	N	n(%)	
Ulcer	10	10 (100.0)	25	18 (72.0)	0.084
Number of ulcers					
1	10	1 (10.0)	18	3 (11.1)	1.000
≥ 2	10	8 (80.0)	18	15 (72.2)	
Unknown	10	1 (10.0)	18	0 (0.0)	
Depth of ulcer					
Deep	10	0 (0.0)	18	8 (44.4)	0.003
Superficial	10	10 (100.0)	18	5 (27.8)	
Unknown	10	0 (0.0)	18	5 (27.8)	
Ulcer located at stricture site	10	9 (90.0)	18	1 (5.6)	0.000
Strictures	10	10 (100.0)	25	18 (72.0)	0.084
1	10	2 (20.0)	18	7 (38.9)	0.417
≥ 2	10	8 (80.0)	18	11 (61.1)	
Cobblestone appearance	10	0 (0.0)	25	2 (8.0)	1.000
Transmural inflammation	10	1 (10.0)	25	16 (64.0)	0.007
Non- caseous epithelioid granulomas	10	0 (0.0)	25	1 (4.0)	1.000

*CMUSE* cryptogenic multifocal ulcerous stenosing enteritis; *SBCD* small bowel Crohn’s disease

## Discussion

Our study showed the following similarities between CMUSE and SBCD: (1) Both diseases had a chronic and recurrent course; (2) Abdominal pain was the most common reporting and persistent symptom in both entities; (3) Both diseases were associated with extra-intestinal manifestations such as oral ulcers; (4) Anemia and hypoalbuminemia frequently occurred in both diseases; (5) Positive ASCA was present in both CD and CMUSE [12]. (5) Lesions of CMUSE may be separated by normal mucosa, mimicking skip lesions of CD. (6) Both diseases most commonly involved ileum [13]. Although rare, CMUSE can affect duodenum and ileocecal regions, consistent with previous reports [14]. (7) Intestinal bleeding and obstruction were characteristic for both SBCD and CMUSE. Given these similarities, it is often difficult to distinguish CMUSE from SBCD based on clinical, radiographic, and endoscopic features [5]. In fact, half of CMUSE patients in our cohort had been misdiagnosed with CD before the correct diagnosis was made.

Beyond these similarities, however, our study did reveal some useful clues to distinguish these two diseases. First, hematochezia (71.4% vs 37.7%) was nearly two times more often in CMUSE than in SBCD, while diarrhea was present in about one third patients with SBCD but was absent in CMUSE. Intestinal strictures were universally present in all CMUSE patients but only occurred in about two third of patients with SBCD. Intraabdominal fistula, resulting from deep transmural ulcer, was considered to be diagnostic of CD [15]. But in this study the incidence of fistula in CMUSE and SBCD patients had no significant differences (0.0% vs 11.5%).. According to current literature [11], only 15.5% of CD patients have penetrating lesions (fistulas, phlegmons or abscesses) at the time of diagnosis. Limited sample size and low incidence of fistula may explain the lacking of statistical significance in this study.

Our study confirmed that serum inflammatory markers such as ESR and hsCRP elevated more often in SBCD than in CMUSE patients. For example, ESR was normal in all cases of CMUSE, consistent with another study that enrolled 17 CMUSE patients in France [10]. In contrast, ESR was elevated in half of SBCD patients and hsCRP in about two thirds. High hsCRP in 28.6% CMUSE patients in our study may results from inflammatory response following acute exacerbation of small bowel obstruction. CTE is widely used for the diagnosis, evaluation and surveillance of small bowel lesions. Our study confirmed that extra-enteric findings, such as enlarged intraabdominal lymph nodes, were significantly more common in SBCD patients. These findings should remind clinicians that extra-luminal manifestations on radiographic examination are useful in differentiating CMUSE from SBCD [2, 16]. Endoscopy allows for direct visualization and biopsy for small bowel lesions. In our study a vast majority of both CMUSE and SBCD patients underwent at least once endoscopic examination. Double-balloon enteroscopy plays an essential role in the diagnosis of CMUSE and SBCD. Ulcer morphology and number of strictures detected by endoscopy helps to discriminate CMUSE and SBCD. Consistent with the literature [16, 17], longitudinal ulcer (37.2% vs 0.0%) was diagnostic for SBCD patients, while CMUSE patients more often developed circumferential ulcer (54.6% vs 18.6%) than SBCD.

According to histological examination, CMUSE and SBCD differs in ulcer locations, ulcer depth, and transmural inflammation. Ulcers are located on the sites of stricture in all CMUSE patients, but in SBCD patient ulcers often present at the oral side of strictures, probably due to elevated intraluminal pressure arising from distal obstruction [10]. In terms of depth, ulcers in CMUSE patients were all superficial, limited within mucosal and submucosal layers. Ulcers in SBCD, in contrast, tended to be deep with the most characteristic finding of “fissure-like”. Noteworthy is that the depth of ulcers in SBCD correlates with disease progression, making it possible that superficial ulcers are present in SBCD at early stage. In the SBCD group of this study, nearly half patients presented deep ulcers but about one fourth patients did have superficial ulcers only. Transmural inflammation, a pathognomonic histological finding in CD, occurred more common in SBCD (64.0%) compared with CMUSE (10.0%) patients in our study. Another typical sign of CD, namely non-caseous epithelioid granuloma, was not significant different between the two groups probably due to its low occurrence rate and limited sample size obtained by enteroscopy. Other authors reported non-caseous epithelioid granuloma in only 13 to 36% of patients with CD [18, 19]. Therefore, CD should not be excluded based on lacking of non-caseous epithelioid granuloma due to its low sensitivity.

Significant advances in the pathogenesis of CMUSE has been made recently since an attempt has been made to decode its genetic basis. CMUSE is believed to be an “autosomal recessive” disease resulted from mutation of gene leading to the impaired prostaglandin function such *SLCO2A1* gene [20, 21] and/or *PLA2G4A* gene [14]. Gene mutation analysis, immunohistochemical staining for SLCO2A1 protein in gastroduodenal tissues and prostaglandin E major urinary metabolites (PGE-MUM), a major urinary metabolite derived from PGE2, may help differential those two diseases [22–25]. However, such conclusions could not be made from our study because we failed to get these information. This is one of the limitations of our study. There were several other limitations in our study. Since this study was a retrospective study in a single center, and all patients enrolled were hospitalized, certain selection bias was inevitable. Besides, the number of CMUSE patients enrolled was limited due to the rarity of the disease. Further large multi-center studies are needed to explore the differential markers between these two entries.

## Conclusions

In conclusion, CMUSE and SBCD have overlapping features and discrimination between the two conditions can be difficult. However, some valuable clues are helpful in the differential diagnosis, including gastrointestinal symptoms such as hematochezia, fever and diarrhea, the complications such as intestinal stenosis, terminal ileum involving, anemia, level of serum inflammatory markers, CTE features including extra-enteric findings and enlargement of the abdominal lymph nodes, endoscopic features such as ulcer types and stricture number and pathologic features such as ulcer depth, ulcer location and transmural inflammation.

## Supplementary information

**Additional file 1.**

## Data Availability

The datasets used during the current study are presented in the main manuscript.

## Abbreviations

ANCA: Antineutrophil cytoplasmic antibodies
ASCA: Antisacchromyces cerevisia antibody
BMI: Body mass index
CD: Crohn’s disease
CMUSE: Cryptogenic multifocal ulcerous stenosing enteritis
CTE: Computed tomography enterography
ESR: Erythrocyte sedimentation rate
hsCRP: High sensitivity C reactive protein
NSAIDs: Nonsteroidal anti-inflammatory drugs
PGE-MUM: Prostaglandin E major urinary metabolites
SBCD: Small bowel Crohn’s disease
SD: Standard deviation
WBC: White blood cells
