# Supplementary material for: Retrospective study of the differential diagnosis between cryptogenic multifocal ulcerous stenosing enteritis and small bowel Crohn’s disease
Source: BMC Gastroenterol. 2020 Aug 5;20:252. doi: 10.1186/s12876-020-01389-7 (PMC7409495; doi:10.1186/s12876-020-01389-7)
Supplement: Supplementary file 1 — Additional file 1. [file 12876_2020_1389_MOESM1_ESM.docx]

**Supplementary Data Content 1: Laboratory findings of CMUSE and SBCD patients**

| Characteristics | CMUSE | | SBCD | | *P* value |  |
| --- | --- | --- | --- | --- | --- | --- |
|  | N | n(%) | N | n(%） |  | |
| Elevated WBC | 14 | 3(21.4) | 60 | 10(16.7) | 0.954 | |
| Anemia | 14 | 13(92.9) | 60 | 36(60.0) | 0.043 | |
| Microcytic hypochromic Anemia | 13 | 7(53.9) | 36 | 14(38.9) | 0.350 | |
| Normocytic anemia | 13 | 6(46.2) | 36 | 16(44.4) | 0.915 | |
| Macrocytic anemia | 13 | 0(0.0) | 36 | 4(11.1) | 0.507 | |
| Unknown | 13 | 0(0.0) | 36 | 2(5.6) | / | |
| Fecal occult blood positive | 14 | 12(92.9) | 59 | 40(67.8) | 0.120 | |
| Elevated ESR | 13 | 0(0.0) | 60 | 30(50.0) | 0.001 | |
| Elevated hsCRP | 14 | 4(28.6) | 57 | 39(68.4) | 0.006 | |
| Hypoalbuminema | 14 | 4(28.6) | 60 | 33(55.0) | 0.075 | |
| ANCA positive | 12 | 1(8.3) | 52 | 2(3.7) | 0.470 | |
| ASCA positive | 11 | 3(27.3) | 33 | 8(24.2) | 1.000 | |

CMUSE: cryptogenic multifocal ulcerous stenosing enteritis; SBCD: small bowel Crohn’s disease; WBC: white blood cells; ESR: erythrocyte sedimentation rate; hsCRP: high-sensitivity C-reactive protein; ANCA: antineutrophil cytoplasmic antibodies; ASCA: antisacchromyces cerevisia antibody.

**Supplementary Data Content 2: Surgical operation data of CMUSE and SBCD patients**

| Characteristics | CMUSE | | SBCD | | *P* value |
| --- | --- | --- | --- | --- | --- |
|  | N | n(%） | N | n(%） |  |
| Proportion of patients underwent surgery | 14 | 10(71.4) | 61 | 25（41.0） | 0.018 |
| Surgical indication |  |  |  |  |  |
| Ileus | 10 | 6(60.0) | 25 | 12（48.0） | 0.711 |
| Hematochezia | 10 | 1(10.0) | 25 | 5（20.0） | 0.649 |
| Abdominal mass | 10 | 0(0.0) | 25 | 4（16.0） | 0.303 |
| Intestinal stenosis without ileus | 10 | 1(10.0) | 25 | 2（8.0） | 1.000 |
| Capsule endoscopy retention | 10 | 2(20.0) | 25 | 1（4.0） | 0.190 |
| Gastrointestinal perforation | 10 | 0(0.0) | 25 | 1（4.0） | 1.000 |

CMUSE: cryptogenic multifocal ulcerous stenosing enteritis; SBCD: small bowel Crohn’s disease.
